# A model-based quantification of startle reflex habituation in larval zebrafish

**DOI:** 10.1038/s41598-020-79923-6

**Published:** 2021-01-12

**Authors:** Carolina Beppi, Dominik Straumann, Stefan Yu Bögli

**Affiliations:** 1 Neuroscience Center Zurich, University of Zurich and ETH Zurich, CH-8091 Zurich, Switzerland; 2 Department of Neurology, University Hospital Zurich and University of Zurich, CH-8091 Zurich, Switzerland; 3 Clinical Neuroscience Center, University Hospital Zurich and University of Zurich, CH-8091 Zurich, Switzerland; 4 Swiss Concussion Center, Schulthess Clinic, CH-8008 Zurich, Switzerland

**Keywords:** Physiology, Neuroscience, Auditory system, Neuronal physiology, Sensorimotor processing

## Abstract

Zebrafish is an established animal model for the reproduction and study of neurobiological pathogenesis of human neurological conditions. The ‘startle reflex’ in zebrafish larvae is an evolutionarily preserved defence response, manifesting as a quick body-bend in reaction to sudden sensory stimuli. Changes in startle reflex habituation characterise several neuropsychiatric disorders and hence represent an informative index of neurophysiological health. This study aimed at establishing a simple and reliable experimental protocol for the quantification of startle reflex response and habituation. The fish were stimulated with 20 repeated pulses of specific vibratory frequency, acoustic intensity/power, light-intensity and interstimulus-interval, in three separate studies. The cumulative distance travelled, namely the sum of the distance travelled (mm) during all 20 stimuli, was computed as a group-level description for all the experimental conditions in each study. Additionally, by the use of bootstrapping, the data was fitted to a model of habituation with a first-order exponential representing the decay of locomotor distance travelled over repeated stimulation. Our results suggest that startle habituation is a stereotypic first-order process with a decay constant ranging from 1 to 2 stimuli. Habituation memory lasts no more than 5 min, as manifested by the locomotor activity recovering to baseline levels. We further observed significant effects of vibratory frequency, acoustic intensity/power and interstimulus-interval on the amplitude, offset, decay constant and cumulative distance travelled. Instead, the intensity of the flashed light did not contribute to significant behavioural variations. The findings provide novel insights as to the influence of different stimuli parameters on the startle reflex habituation and constitute a helpful reference framework for further investigation.

## Introduction

Zebrafish has become an established animal model due to several advantages, including its small size, the prosperous offspring, the rapid development through defined stages, the simple breeding and the easy maintenance of large stocks^1,2^. The existence of established microscopic imaging approaches^3–8^ and the optical translucency until larval stages^1,6,7^ allow detailed non-invasive tracking of CNS changes following specific neural manipulations or chemical interventions. Cell-manipulations (e.g., in Bhandiwad et al.^9^) and electrophysiological recordings can assess the zebrafish sensory-processing functions fast and reliably^10–13^, although invasively. Behavioural screenings are therefore becoming preferred assessment-methods of sensory-processing and attention^14^.

### Habituation of the startle reflex: a basic form of learning

Zebrafish at larval stage already exhibit a vast behavioural repertoire that can be reliably tracked and consistently quantified^15–19^. First probed in the 1970s^20^, the startle reflex in zebrafish larvae is a fast body muscle contraction in response to abrupt sensory stimuli^3,21,22^ and is an evolutionarily preserved mechanism of defence against threat. It is a ‘C-bend’ of the body^23^ largely mediated by the Mauthner cells (MC), a bilateral pair of large reticulospinal interneurons in the zebrafish hindbrain^22–26^. They receive input ipsilaterally, through their lateral dendrites, from sensory receptors of different modalities^24–27^, although the mechanosensory hair cells are the main driver^24,26^.

Transient light stimuli evoke increased escape responses kinematically identical to acoustic startle reflex (ASR) C-bends and proportional to the magnitude of change in lighting^21^, but of a longer latency (M =  ~ 183 ms) compared to acoustic and touch-evoked (M ~ 15 ms) startle responses^21^. The startle reflex can also be non-MC initiated, although it has a slower onset^28^. Startle responses are modulable by internal factors like social dominance (in adult fish), arousal or stress^29–31^. Increase in the startle reflex can be induced by sensitisation, fear-potentiation^32^, and pharmacological compounds affecting the methyl-D-aspartic acid signalling^33,34^, while decremented ASR can be caused by pre-pulse inhibition^35,36^ and by habituation^35^.

Habituation is a basic, non-associative, form of learning defined by the cessation of an innate response after repeated or sustained exposure to a sensory stimulus^28,37,38^. It has been studied in several invertebrate species, including Aplysia, Tritonia, Drosophila, as well as rodents, with a variety of stimuli^39–42^. The habituation of the startle reflex in larval zebrafish has been probed in the 1970s^24,43^. It is consistently elicitable and quantifiable, although non-stereotypic^29–31^. The amount of stimulation the fish is exposed to determines the endurance of habituation^28^. Specifically, three types of habituation persistence have been described: rapid habituation (≤ 15 min) is induced by low-frequency stimuli, while short-term habituation (≤ 1 h) can be induced by a few blocks of stimuli. If the blocks are repeated over several sessions, habituation can also endure for days (long-term habituation).

Changes in startle reflex and habituation have been reported in neuropsychiatric disorders, including schizophrenia^44^, traumatic brain injury and post-traumatic stress disorder^45,46^. The startle reflex and habituation are thus becoming established neurophysiological indices for clinical and translational investigation in neurology and psychiatry.

### Aims and hypotheses

A number of studies report that the number, duration and intensity of the stimuli, as well as the inter-stimuli interval and the number of blocks or sessions can affect the profile of the startle reflex habituation^19,28,35,47–49^*.* However, the considerable variability in the experimental design and analytical approach of existing studies do not allow a direct comparison between the findings, preventing the possibility to draw conclusions about the optimal experimental parameters. The acquisition of complex data is one of the factors that may hinder the replicability and reproducibility of the findings, which is a recurrent issue in behavioural (neuroscience) research in zebrafish^50^. This investigation aimed at defining a novel experimental protocol for the study and assessment of startle reflex and its habituation. Specifically, we aimed at designing a simple behavioural paradigm that could be more easily reproducible and adaptable for testing different experimental questions, thus allowing to understand the influence of specific experimental parameters on the profile of the startle reflex and its habituation. We investigated startle reflex habituation in three separate studies, each manipulating and assessing the isolated effect of different stimulus features on the profile of the startle reflex (acoustic or visual) habituation.

A first study (1) investigated the time necessary for the zebrafish to extinguish the habituation, quantifying the ASR habituation at different re-test times relative to the baseline test. Based on Roberts et al.^28^, we hypothesised that startle extinction of ASR habituation memory, quantified as the mean of all fish's cumulative distance travelled (CDT) during all 20 stimuli, would be significantly different from baseline, at the different re-test times (1, 5 or 15 min). The second study (2) investigated the main effect of vibratory power (a), vibratory frequency (b), interstimulus interval (ISI) duration (c) on the profile of the ASR habituation. The third study (3) assessed the effect of light-flash illuminance on the profile of the visual startle reflex (VSR) habituation. Based on previous findings^19,28,35,47–49^, we further hypothesised that startle reflex habituation would differ significantly between vibratory frequency conditions, acoustic power, ISI duration and illuminance.

## Results

### Study 1—Extinction of ASR habituation memory

This study was conducted to determine the decay of habituation memory, namely the time necessary for the fish to ‘lose memory’ of the stimulus, as manifested by the locomotor responses to the stimulus returning up to baseline levels. It was hypothesised that in each re-test time group, there would be a statistically significant difference in average CDT at re-test compared to baseline.

**Figure 1:**
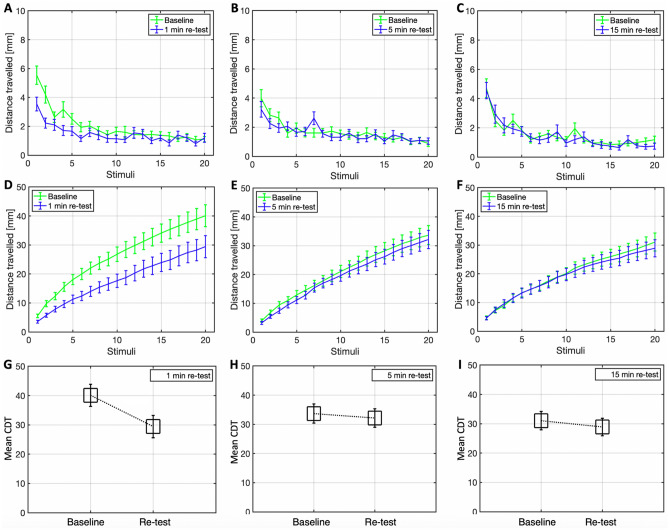
Plots of the distance travelled (M ± 1 SE) at each stimulus, and cumulatively over successive stimuli at baseline and re-test, for the 1 min (**A**, **D**), 5 min (**B**, **E**) and 15 min (**C**, **F**) re-test groups. Boxplots for the CDT (M ± 1 SE) between the baseline and re-test conditions of the 1 min (**G**), 5 min (**H**) and 15 min (**I**) re-test group.

Wilcoxon signed-rank t-tests (N = 48) indicated that the median CDT rank at baseline was significantly higher compared to at 1 min re-test (Z = 202, p < 0.001), but not at 5 min re-test (Z = 469, p = 0.58) or at 15 min re-test (Z = 468, p = 0.31), as shown in Fig. 1G–I. The mean distance travelled at each stimulus, by each group, is depicted in Fig. 1A–F. Descriptive statistics for the mean CDT values of all groups are reported in Table 1 (study 1).

**Table 1:** Descriptive statistics for the mean [M] and standard error [SE] of the CDT (mm) of all the groups in all the studies.

	Study 1							
	1 min		5 min		15 min			
	*N* = 48		*N* = 48		*N* = 48			
	M	SE	M	SE	M	SE		
Baseline	40.05	3.77	33.7	3.28	31.07	3.12		
Re-test	29.43	3.8	32.16	3.18	28.9	3.0		

	Study 2a		Study 2b		Study 2c		Study 3	
	*N* = 90		*N* = 120		*N* = 120		*N* = 96	
	M	SE	M	SE	M	SE	M	SE
Control	6.96	1.25	9.4	1.02	8.03	0.78	13.47	1.17
Condition 1	19.65	1.96	10.44	1.23	33.64	2.68	23.13	1.86
Condition 2	15.41	1.69	11.95	1.29	34.53	2.53	21.63	1.76
Condition 3	10.75	1.44	16.28	1.6	41.1	3.12	21.4	1.97
Condition 4	8.59	1.31	26.91	2.16	51.96	3.31	–	–

The response probability was: 85% and 75% for the 1 min baseline and re-test conditions respectively, 77% and 67% for the 5 min baseline and re-test conditions respectively, 87% and 77% for the 15 min baseline and re-test conditions respectively.

### Study 2—Effect of vibratory frequency (a), power (b) and ISI duration (c) on the ASR habituation

#### 2a

**Figure 2:**
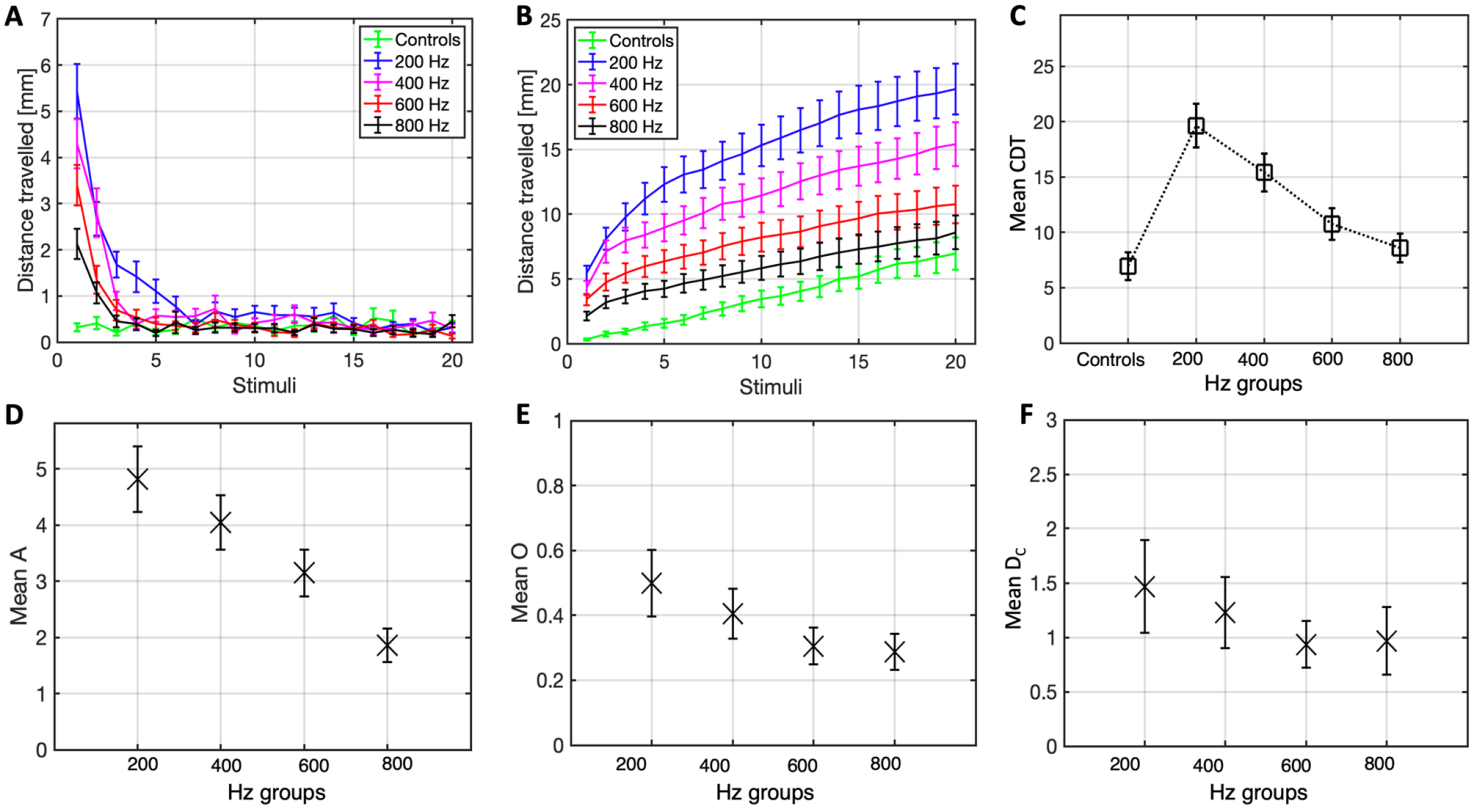
Plots of the distance travelled (M ± 1 SE) at each stimulus (**A**), cumulative distance travelled over successive stimuli (**B**), and mean CDT boxplots (**C**) for all the frequency conditions and the control group. Boxplots for the amplitude (**D**), offset (**E**) and decay constant (**F**) of 250 bootstraps (M and SE), for all frequency conditions.

The next study was conducted to assess the main effect of vibratory frequency on the ASR habituation. It was first hypothesised that there would be a statistically significant difference in average CDT between the groups. A Friedman’s test (N = 90) indicated an overall statistically significant difference in mean CDT rank between the frequency conditions (χ^2^(4) = 90.187, p < 0.001), as shown in Fig. 2C. Significant differences at the pairwise level are reported in Table 2. Descriptive statistics for the mean CDT values of all groups are instead reported in Table 1.

**Table 2:** Statistics with Bonferroni adjustments for the significant differences at pairwise level of studies 2 and 3.

Study 2a		Study 2b		Study 2c		Study 3	
	Adj. Sig.		Adj. Sig.		Adj. Sig.		Adj. Sig.
Control Vs Condt. 1	< 0.001	Condt. 4 Vs Control	< 0.001	Control Vs Condt. 1	< 0.001	Control Vs Condt. 1	< 0.001
Control Vs Condt. 2	< 0.001	Condt. 4 Vs Condt. 1	< 0.001	Control Vs Condt. 2	< 0.001	Control Vs Condt. 2	< 0.001
Control Vs Condt. 3	0.015	Condt. 4 Vs Condt. 2	< 0.001	Control Vs Condt. 3	< 0.001	Control Vs Condt. 3	< 0.001
Condt. 4 Vs Condt. 1	< 0.001	Condt. 4 Vs Condt. 3	0.02	Control Vs Condt. 4	< 0.001		
Condt. 4 Vs Condt. 2	0.002			Condt. 4 Vs Condt. 1	0.002		
Condt. 3 Vs Condt. 1	< 0.001			Condt. 4 Vs Condt. 2	0.009		

**Figure 3:**
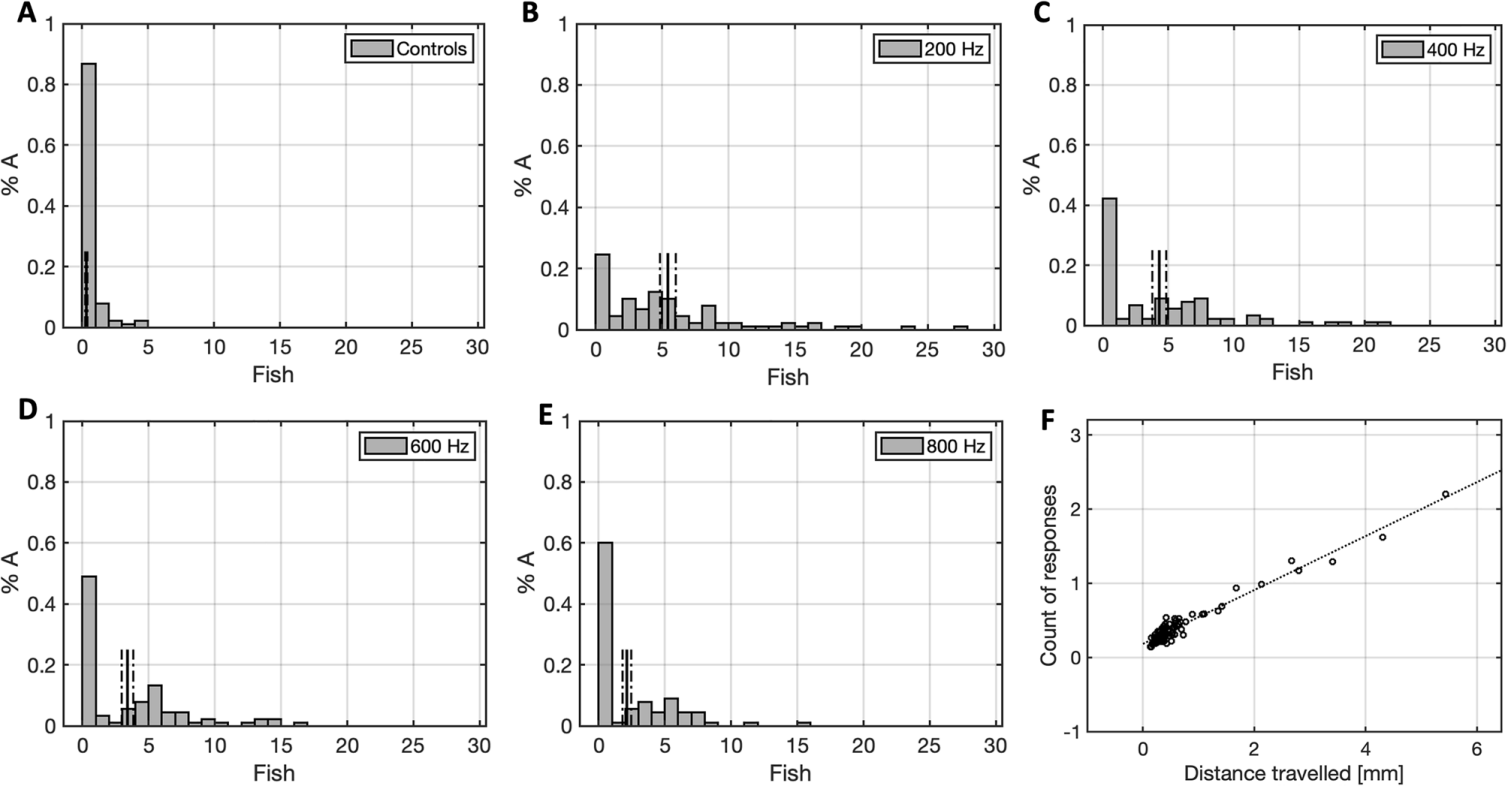
(**A**–**E**) Histograms of the percentage (%) amplitude (M [black line] ± 1 SE [dotted line]) in each of the experimental conditions. (F) Relationship, with line of best fit, between the fish mean count of responses and mean distance travelled, at each stimulus, for all frequency conditions.

It was secondly hypothesised that the discrete and continuous measures in all the frequency conditions would show a statistically significant association. The designed continuous measure (distance travelled, mm) and discrete measure (count of responses) reliably quantified the ASR habituation, showing a strong positive Spearman correlation (see Fig. 3F), statistically significant in all the experimental conditions (200 Hz: r = 0.94, p < 0.001; 400 Hz: r = 0.71, p < 0.001; 600 Hz: r = 0.89, p < 0.001; 800 Hz: r = 0.74, p < 0.001), but not in the control (r = 0.35, p = 0.13).

The mean distance travelled of all groups is presented in Fig. 2A,B. The response probability for the respective conditions were 21% (controls), 80% (200 Hz), 62.2% (400 Hz), 60% (600 Hz), 45.5% (800 Hz). The mean amplitude (A), offset (O) and decay constant (D_C_) for the bootstrapped (N = 250) distance travelled of all fish in each group is illustrated in Fig. 2D–F.

Histograms highlighting the distribution of the sample’s amplitude are shown in Fig. 3A–E.

#### 2b

**Figure 4:**
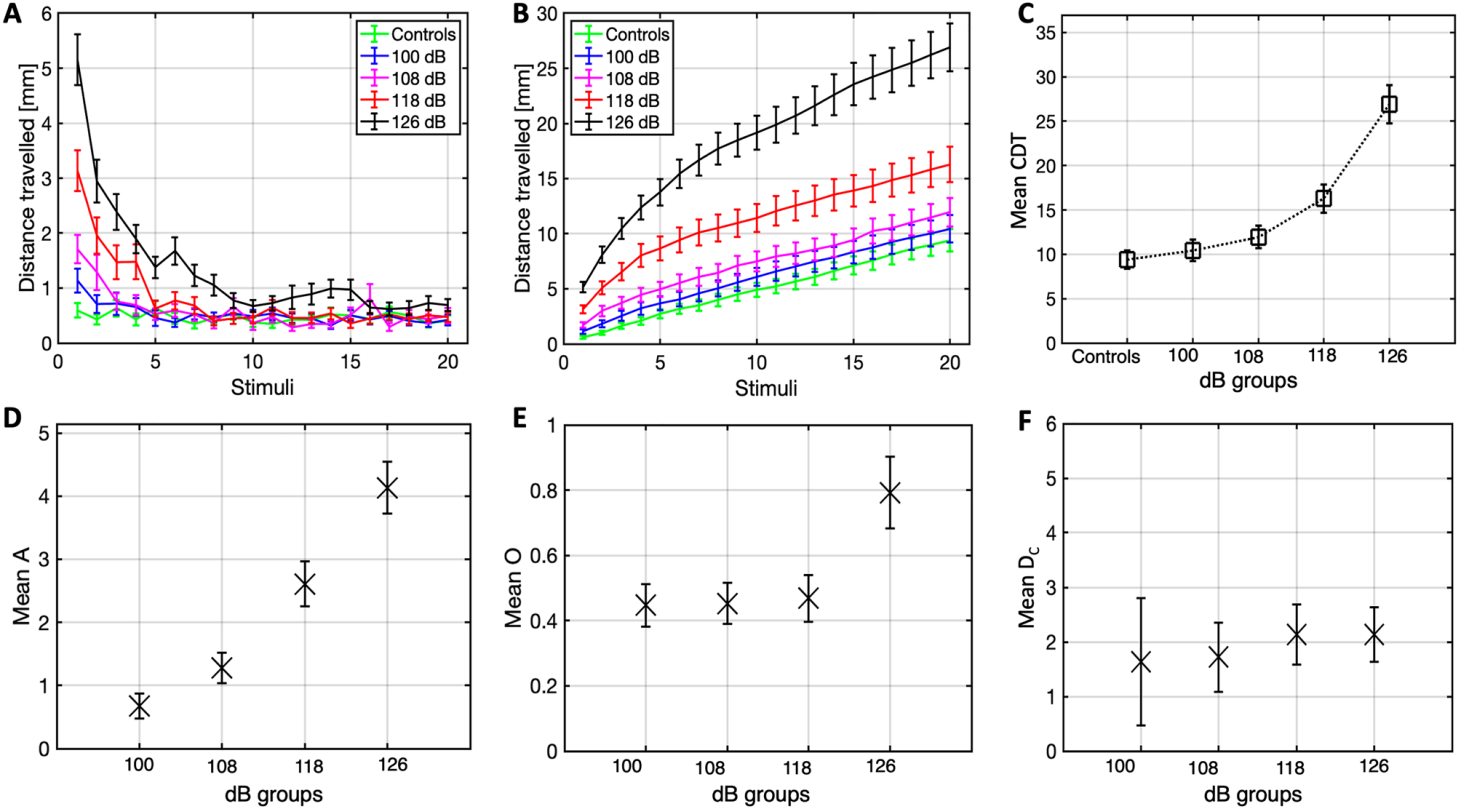
Plots of the distance travelled (M ± 1 SE) at each stimulus (**A**), cumulative distance travelled over successive stimuli (**B**), and mean CDT boxplots (**C**) for all the acoustic power groups and the control group. Boxplots for the amplitude (**D**), offset (**E**) and decay constant (**F**) of 250 bootstraps (M and SE), for all acoustic power conditions.

The next study was conducted to assess the main effect of acoustic power on the ASR habituation. It was hypothesised that there would be a statistically significant difference in average CDT between the power groups. A Friedman’s test (N = 120) revealed a significant difference in mean CDT rank between the groups (χ^2^(4) = 30.89, p < 0.001), as shown in Fig. 4C. Significant differences at the pairwise level are reported in Table 2. Descriptive statistics for the mean CDT value of all groups are provided in Table 1.

The mean distance travelled of all groups is presented in Fig. 4A,B. The response probability for the respective conditions were 28.3% (controls), 34.2% (100 dB), 44.2% (108 dB), 53.3% (118 dB), 65% (126 dB). The mean amplitude, offset and decay constant for the bootstrapped (N = 250) distance travelled of all fish in each group is illustrated in Fig. 4D–F.

#### 2c

**Figure 5:**
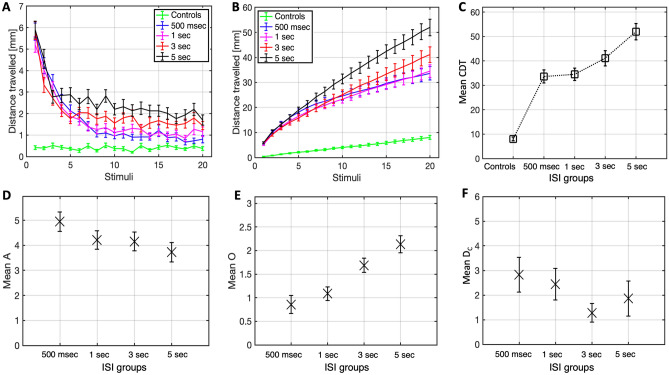
Plots of the distance travelled (M ± 1 SE) at each stimulus (**A**), cumulative distance travelled over successive stimuli (**B**), and mean CDT boxplots (**C**) for all the ISI duration groups and the control group. Boxplots for the amplitude (**D**), offset (**E**) and decay constant (**F**) of 250 bootstraps (M and SE), for all ISI duration conditions.

The next study was conducted to assess the main effect of ISI duration on the ASR habituation. It was hypothesised that there would be a statistically significant difference in average CDT between the ISI groups. A Friedman’s test (N = 120) revealed a significant difference in mean CDT rank between the groups (χ^2^(4) = 113.497, p < 0.001), as shown in Fig. 5C. Significant differences at the pairwise level are reported in Table 2. Descriptive statistics for the mean CDT values of all groups are provided in Table 1.

The mean distance travelled of all groups is presented in Fig. 5A,B. The response probability for the respective conditions were 30% (controls), 90.8% (500 ms), 87.5% (1 s), 83.3% (3 s), 86.7% (5 s). The mean amplitude, offset and decay constant for the bootstrapped (N = 250) distance travelled of all fish in each group is illustrated in Fig. 5D–F.

### Study 3—Illuminance of light-flash in the VSR

**Figure 6:**
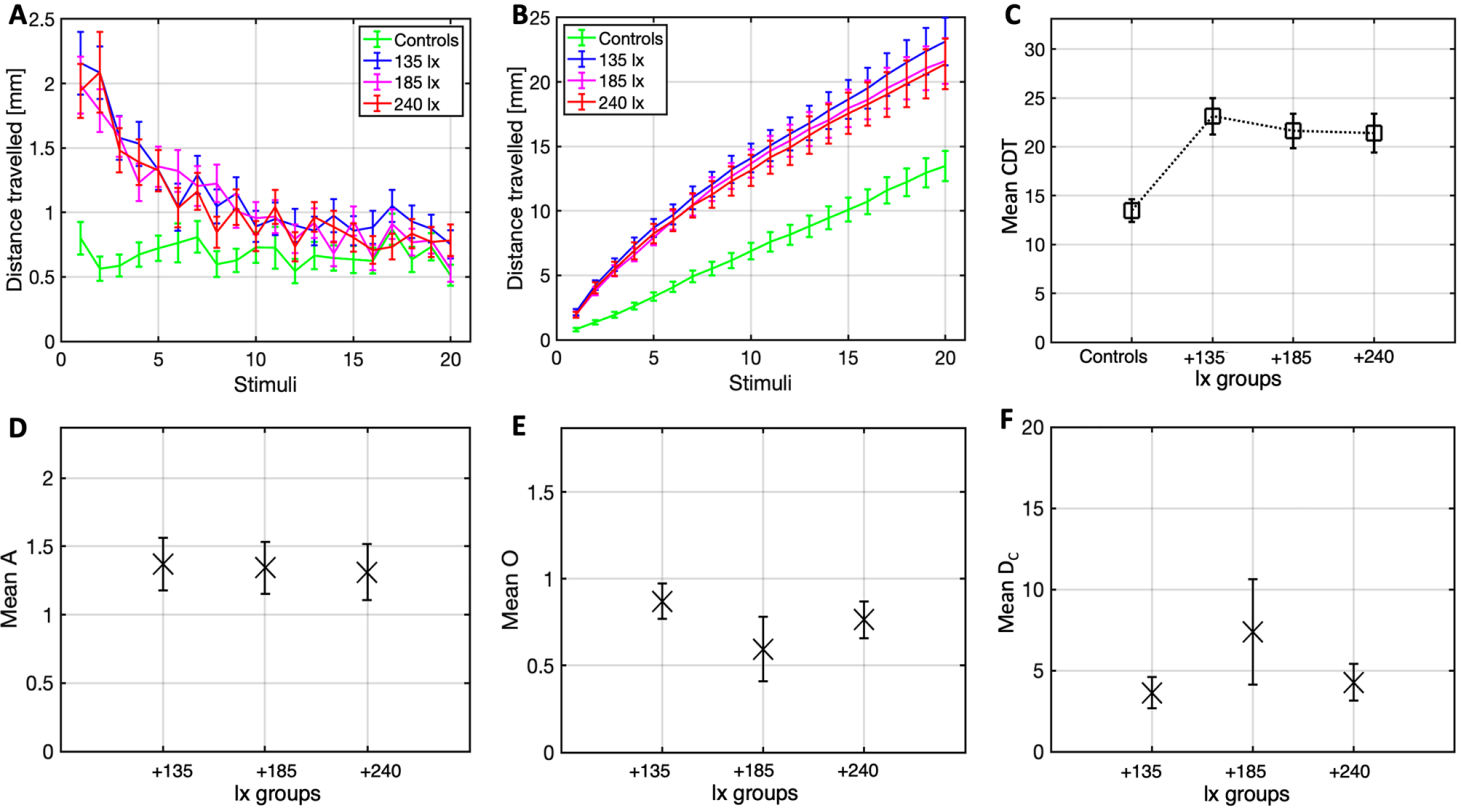
Plots of the distance travelled (M ± 1 SE) at each stimulus (**A**), cumulative distance travelled over successive stimuli (**B**), and mean CDT boxplots (**C**) for all the illuminance groups and the control group. Boxplots for the amplitude (**D**), offset (**E**) and decay constant (**F**) of 250 bootstraps (M and SE), for all the illuminance conditions.

This third study was conducted to assess the main effect of illuminance of white light-flashes on the VSR habituation. It was hypothesised that there would be a statistically significant difference in average CDT between the illuminance groups. A Friedman’s test (N = 96) revealed a significant difference in mean CDT ranks between the groups (χ^2^(3) = 48.754, p < 0.001), as shown in Fig. 6C. Significant differences at the pairwise level are reported in Table 2. Descriptive statistics for the mean CDT values of all groups are provided in Table 1.

The mean distance travelled of all groups is presented in Fig. 6A,B. The response probability for the respective conditions were 56.3% (controls), 68.8% (135 lx), 65.6% (180 lx), 63.5% (240 lx). The mean amplitude, offset and decay constant for the bootstrapped (N = 250) distance travelled of all fish in each group is illustrated in Fig. 6D–F.

## Discussion

Changes in startle response and habituation are reported in several neuropsychiatric disorders and thus constitute a relevant neurophysiological index for clinical and translational research. The startle reflex habituation is a consistently elicitable response, although non-stereotypic, that can be reliably quantified in larval zebrafish. A number of different stimuli properties have been shown to influence its profile, including the number, duration, intensity of stimuli and inter-stimuli intervals as well as the numbers of blocks and sessions. However, the inconsistency between the experimental designs insofar have prevented a direct comparison between the findings and the extrapolation of systematic conclusions.

Our investigation therefore aimed at probing a novel analytical approach for the study and assessment of sample’s startle habituation through average CDT quantifications, by using a simpler behavioural paradigm (a few stimuli, single block), for a matter of more simplicity, clarity and better control. Simple paradigms have the advantage of being more adaptable for testing different experimental hypotheses and of being more easily and reliably replicable by other researchers, in turn generating more reproducible findings. Average locomotion distances over the 20 stimuli have been fitted with a first-order exponential decay function, to extract three measures: amplitude, offset and decay constant. The standard error of the three measures was computed by 250 bootstraps of each study’s data, separately for each experimental condition. So far, mathematical models of behavioural quantification in larval zebrafish have only been applied in the ocular motor control fields (e.g., Ulrich et al.^51^). To our knowledge, no existing study has applied exponential fitting for describing the startle reflex habituation.

Using a simple experimental protocol and descriptive model of behaviour, this study has for the first time systematically assessed the effect of different stimuli properties on the profile of the ASR and VSR habituation. We specifically tested the influence of vibratory frequency and power, the ISI duration and light-flash illuminance on the ASR and VSR habituation, as well as the extinction of ASR habituation memory, in four separate studies. It was hypothesised that the average CDT would be significantly different among re-test times (1, 5, 15 min), relative to baseline. It was additionally hypothesised that the CDT would be statistically significantly different between vibratory frequency and power, ISI duration, as well as light-flash illuminance conditions.

The results of the study 1 highlight a significantly lower CDT at 1 min re-test, compared to baseline. This suggests that the fish withhold memory of the startle habituation one minute after exposure, and that the memory markedly affects the vigorousness of the responses to the first few stimulations. The responsivity seems instead to be mostly unaffected, evidenced by a weak reduction in response probability at re-test, relative to baseline. No differences in the habituation profile have been found between baseline and re-test after 5 and 15 min, suggesting that habituation memory relapses within 5 min. The findings demonstrate that 20 stimuli of high frequency (400 Hz) can give rise to a form of rapid habituation (< 15 min) that is similar to that achieved by Roberts et al.^28^ with several (< 100) low-frequency stimuli (0.5–2 Hz).

One might notice that the three groups had differing baseline motility levels. In this regard, we expected that different samples (groups) of fish might show differing baseline behaviour due to individual differences in the intrinsic vigorousness of motor responses (see Portugues and Engert^52^), which might bias the average measures of certain samples, relative to the population. In addition, developmental differences between fish of 4–5 days are also plausible and may affect a specific sample’s baseline behaviour. It follows that the most reliable types of comparisons are within-group analyses (repeated-measures t-tests comparing baseline with re-test, for each group), which we have conducted. In order to make intergroup comparisons (e.g., in average CDT), one would need to run an analysis of “deltas”, considering the change in CDT at re-test relative to baseline, and testing it for differences between the three groups, by mean of an independent-measures ANOVA. This would exclude/discard eventual differences in baseline motility levels of the different groups. By running such independent-measures analysis, we would expect similar findings, with the change (reduction) in motility at re-test relative to the respective baseline level, being significantly higher at 1 min re-test compared to when at 5 min or 15 min re-rest.

The results of study (2a) revealed that vibratory frequency has a significant effect over the ASR habituation. A significantly lower CDT was found when the fish were tested with 800 or 600 Hz stimuli, compared to when exposed to 200 or 400 Hz stimuli. When tested with 200 Hz in particular, the fish responded more vigorously to the first few stimulations, relatively to the other conditions. Our findings support Bhandiwad et al.^35^, suggesting that ~ 200 Hz is the frequency inducing the most robust ASR. Interestingly, a general trend of linear decrease in distance travelled from lower to higher frequencies can be observed. In line with this, response probability diminished with higher frequencies, reducing from 80% (200 Hz) to 45.5% (800 Hz). This is further highlighted by the truncated distribution of the sample’s amplitude, with an increasing amount of failed (0) responses from lower to higher frequencies (see Fig. 3). A statistically significant markedly positive correlation between the distance travelled and the count of responses provided support on the reliability of the quantitative measures of CDT.

Study (2b) investigated the effect of acoustic power on the ASR habituation. The results revealed an overall significantly higher average CDT for the 126 dB group compared to all the lower power conditions and the control group. A trend of increased CDT along with higher acoustic power was observable. The response probability computations support these observations indicating an increase in reactivity as a function of higher acoustic power. The population-level graphs further highlight that stimuli of higher acoustic intensity affect the amplitude and the offset, which were clearly the highest for the 126 dB group, but not the decay constant of habituation memory, which instead is (arguably) constant across groups. This study has for the first time reported a significant isolated effect of varying acoustic power on the startle reflex habituation, showing that higher vibratory/acoustic intensity is preferable for inducing a robust startle reflex.

Study (2c) explored the effect of varying ISI duration on the ASR habituation. The largely equal response probability across experimental conditions seem to suggest that the responsivity of the fish to acoustic stimulation is unaffected by the ISI duration. However, the statistical results highlight that the lower CDT of the control group relative to all the experimental conditions increases in size and statistical power as a function of longer ISI. Consistently, the 5 s ISI group, followed by the 3 s ISI group, had a significantly higher CDT compared to the 500 ms and 1 s ISI groups. However, a close inspection of the plots in Fig. 5A reveals that the 500 ms- and 1 s ISI groups distance travelled decreased comparably to control levels by the 8th stimulus; a point from which the slope of the cumulative distance-travelled line flattens out in parallel with the control group, suggesting effective habituation. In contrast, the 3 s ISI group, and 5 s ISI group particularly, showed a poor habituation, highlighted by the continued steepness and ‘non-flattening’ cumulative distance travelled line, which explains the higher CDT in the 3 s ISI group, and in the 5 s ISI group, especially.

The population-level graphs confirm that for shorter ISI groups, the amplitude is higher, the offset is smaller and decay constant is larger. An interpretation of the findings would require a conceptual definition between two similar processes: sensory adaptation and habituation. The two terms have often been used interchangeably (e.g., Quon et al.^53^) in that they both cause reduced responses to sensory stimuli, despite the neurobiological substrates are different. Sensory adaptation occurs when a salient sensory stimulus, after repeated or continued exposure, eventually saturates the sensitivity of transducing cells (i.e., adjusted sensory threshold), annulling their firing^54–58^. This would cause a sensory stimulus to be no longer noticeable. As opposed to adaptation, habituation occurs ‘centrally’, namely downstream from the primary sensory transduction^59,60^, such as in the spinal cord^61^ or brainstem for reflex arcs^62^. This means, the behavioural response to the stimulus ceases/reduces, despite it being still processed at primary level. Sensory adaptation and habituation have been studied in several organisms through different sensory triggers. Pulsed or continued pheromone exposure can cause both short-term sensory adaptation and long-term central nervous system (CNS) habituation in grape berry moths^63^ and cabbage loopers^64^ as measured by electroantennogram (EAG) amplitudes. Reiterative visual threat/danger stimuli can reduce escape responses in crabs^65^ and mosquito larvae^66^. Salient olfactory stimuli in humans cause distributed processes at both peripheral and central level in both drosophilae^67^ and humans^68^. Evidence on a functional dissociation between these two processes is still elusive.

The results of study (2c) suggest that a component of sensory adaptation may be contributing to the saturation/flattening of responses observable in the 500 ms and 1 s ISI groups plots. If the stimulations are interleaved by an excessively long ISI, the sensory memory would have already decayed by the onset of the successive stimulus, preventing sensory adaptation; this would justify the steady/continued responsivity of the fish in the in the 3 s and 5 s ISI groups beyond the second half of the stimulations, as highlighted in the distance travelled plots. This hypothesis is supported by the population-level bootstrap analysis, which highlights an increasing offset over longer ISI durations groups. Strikingly, the population-level D_C_ seems to be longer for the 3 s and 5 s ISI groups, compared to the 500 ms and 1 s ISI groups. This observation supports the assumption that a component of sensory adaptation, which may contribute to the faster decay constant in the 500 ms and 1 s ISI groups, is lost if the ISIs are longer than 1 s (i.e., the 3 s and 5 s ISI groups) where only central mechanisms of habituation may contribute to the reduction in responsivity. Sensory cell-recording would be a valuable tool for testing this assumption experimentally. A decreased cell-activity in the 500 ms and 1 s ISI groups, but not in the 3 s and 5 s ISI groups, would confirm the hypotheses. This would be a research question of interest for further study of ASR habituation.

Overall, studies (2) and (3) show that varying the vibratory parameters of the stimuli (e.g., frequency and power/intensity) results in visible changes in amplitude, and null-to-minor effects on the offset, which globally affect the CDT. In contrast, the population-level decay constant seems to be largely unaffected, suggesting that startle habituation with short ISIs (up to 1 s) is a stereotypic first-order process with decay constant ranging from of 1 to 2 stimuli that is consistent regardless of stimulatory parameters.

The 4th and last study investigated the effect of light-flash illuminance on the VSR habituation. As a first consideration for the VSR study, video recordings highlighted a ~ 100 ms slower response onset compared to the ASR studies, in accordance with the past literature^21^. The analyses indicated an overall statistically significant difference in CDT between the conditions, although no pairwise differences between the illuminance conditions were observed. Consistently, despite a visibly smaller CDT of the control group relative to all the experimental groups, an almost equal CDT across the illuminance conditions was observed. Even at population-level, no clear trends are observable for different illuminance conditions. The startle habituation did not vary between illuminance conditions tested.

The absence of differences the VSR habituation across illuminance conditions seems to contradict previous findings^21^, where the frequency of responses of larval zebrafish to a single white-light-flash stimulus of the same length (500 ms) depended on the magnitude-change in lighting. However, differently from their response measure, our amplitude value constitutes a normalised measure, subtracted of the fish-specific offset. Therefore, the normalisation may have cancelled out any differences in absolute reactivity to stimulus 1. This however seems a weak argument, in light of no observable differences in distance travelled at stimulus 1 between illuminance conditions. Other methodological differences which may account for the discrepancy in the findings can however be considered. First, the authors defined their response measures by mean of a movement-decomposition protocol applying precise kinematic criteria, distinguishing ‘scoot’ and ‘R-turn’ initiations, defined as changes in the heading direction by < 30°–40° and > 30°–40°, respectively^69,70^. They observed that while turn initiations increased with the intensity of the light-flash (up to 200), scoot initiations were not significantly altered by the light-flash^21^. As distance travelled in study 1 consisted of a compound measure comprising any movements > 2 mm, including both scoot and R-turn initiations, the global effect size might, in turn, have been deflated. We thus recommend for further research purposes that the visually evoked startle reflex should be quantified separating scoot and R-turn responses.

Second, and perhaps more importantly, in Burgess and Granato’s^21^ study, the fish were pre-adapted at 20 μW cm^−2^ (~ 135 lx) and tested with pulses approaching 200 μW cm^−2^ (~ 1366 lx), corresponding to a final step increase in illuminance of 90%. Instead, in our study, the fish were habituated to a ~ 330 lx baseline-illuminance level and were exposed during the experiment to a step-increase in illuminance of either 35%, 49%, 64%. This means, that the differences between this study and Burgess and Granato’s^21^ study in terms of absolute illuminance (i.e., ~ 1366 lx VS [377 + 240 = 617 lx]) and in terms of baseline-relative step-increase in illuminance (i.e., 90% VS 35%, 49%, 64%) could both account for the discrepancy in the respective study’s findings. Future research might therefore consider re-applying this study’s protocol by using a stronger absolute illuminance or/and a higher baseline-relative step-increase in illuminance, testing whether this results in different findings. Finally, one might argue that the younger age of the larvae in our study (4–5 dpf) relative to Burgess and Granato’s^21^ study (6–7 dpf) could account for a lower acuity and lower discriminatory ability for light-flashes of different illuminance. Nonetheless, this explanation seems unlikely, considering that the zebrafish visual system develops by the 4 dpf^71^ and that no differences in visual behaviour have been found between larvae of 4, 5 and 6 dpf^72^.

Several suggestions for further investigation can be made in relation to the findings of the present study. One might consider varying the parameters used in study 1 and investigate whether high-frequency stimuli other than 400 Hz, different acoustic power levels and shorter/longer ISIs might affect the habituation memory. In light of our findings that 200/400 Hz frequency, 500/1000 ms ISI duration, 126 dB power were the stimuli properties inducing the most robust ASR habituation profile, one might particularly be interested in testing whether this combination of properties would produce a more persistent habituation memory. Interestingly, study 1 showed that habituation memory persisted for at least 1 min and decayed by 5 min, when using ISIs of 1 s. One might thus consider replicating the study using ISIs of 3 s and 5 s, to test whether habituation memory would be less persistent in the 3 s ISI and 5 s ISI conditions (where no sensory adaptation can be achieved) compared to 1 s. Should this hypothesis be confirmed, one could conclude that sensory adaptation contributes to habituation.

Furthermore, one might consider replicating studies (2a) and (2b) exploring higher/lower ranges of vibratory frequency and power. In this regard, we acknowledge the limitation of study (2a) in that the effect of varying acoustic power, demonstrated in study (2b), was not accounted for. Based on study (2b) findings, we suggest that further investigations, wishing to test the effect of different vibratory-frequency ranges, should normalise the frequency conditions for their differences in power.

We finally consider some methodological limitations in this study and discuss aspects that can be improved for further investigation purposes. Startle movements were tracked through an automated detection protocol written in Viewpoint application manager (Viewpoint Life Sciences, Lyon, France), accounting all movements > 2 mm occurring within a 500 ms window. No further processing was performed to discriminate faster (M-cell mediated) by slower (non-M-cell-mediated) C-turns, or to separate C-turns from other types of initiations, including J-turns, O-turns, scoots, burst swims etc.^21,73^. The purpose of such choice was to prioritise and preserve the simplicity of the experimental protocol, thus providing more easily interpretable and reproducible findings. Simpler behavioural paradigms and quantitative methods are, arguably, more versatile and adaptable for testing different experimental questions. Among the experimental protocols used so far for the quantification of *rapid habituation*, we consider ours to be one of the most parsimonious, in that it is extremely simple, yet effective and reliable.

Integrating our findings into a larger perspective, we hope that this study can represent a step forward towards reaching a better replicability and reproducibility of behavioural neuroscience research in zebrafish^50^. We do not discourage future investigations from applying more sophisticated methods of motor-response quantification and decomposition (e.g., Burgess and Granato^21^), which may be able to capture and reveal more fine-grained behavioural aspects in relation to the same experimental manipulations. However, one should consider that this choice may come at the expenses of making the replicability and reproducibility of the findings more difficult. Finally, should one be interested in studying longer-lasting kinds of habituation (*short-term habituation*, *long-term habituation*), more complex paradigms (for a review, refer to Lopez-Schier^29^) would be required.

This paper has provided a novel methodological protocol for a systematic quantification and assessment of acoustic and visually induced startle reflex habituation. Through a simple behavioural paradigm and a mathematical description of response decay, the studies provide novel insights as to the influence of different stimuli properties on the startle responses profile over time. The results have been integrated into a wider context of related findings in the literature, highlighting common grounds and aspects that still require elucidation. We therefore hope that the methodological approach and the conclusions of the current study can provide a framework of reference for research questions and approaches for further investigation in the field.

## Methods

### Ethical approval

All experiments were performed in accordance with the animal welfare guidelines of the Federal Veterinary Office of Switzerland. The animal experiments performed in this study did not require institutional Ethics Committee approval as zebrafish in early larval stages (4–5 dpf) are not protected by Swiss law (TSchV Art. 112).

### Fish maintenance, mating and egg production

Wild-type Tubingen (TU) and Wild Indian Karyotype (WIK) *Danio rerio* adult zebrafish lines were maintained and bred according with standard protocols. Their embryos were raised under a 14-h light, 10-h dark cycle in 28 °C E3 medium (in mM: 5 NaCl, 0.17 KCl, 0.33 CaCl_2_, and 0.33 MgSO_4_; Sigma-Aldrich Corp., St. Louis, MO, USA) and staged according to developmental stage in days post fertilization (dpf). At 3–4 dpf they were transferred to 24-wells plates with E3 solution and maintained there until the experiment occurring the following day.

### Behavioural paradigm

Our novel experimental paradigm consisted of a series of 20 repeated side-wise vibratory stimulations (studies 1 and 2) or light-flashes (study 3) of 500 ms, with instantaneous rise and fall times (square wave). This paradigm was used in all studies and was slightly adapted for testing the different experimental hypotheses. The specific stimuli parameters (i.e., vibratory frequency [Hz], power [dB], ISI duration [ms] and illuminance [lx] chosen or manipulated in each study are detailed in the following sections.

#### Study 1—Extinction of ASR habituation memory

Repeated pulses of 400 Hz had been previously shown to induce a robust ASR^19^. On this basis, 20 vibratory/acoustic stimuli of 400 Hz frequency (126 dB, 500 ms) were chosen for the paradigm. Stimuli were interleaved by ISI of 1 s.

#### Study 2a—Effect of vibratory frequency on the ASR habituation

The paradigm consisted of a series of 20 vibratory/acoustic stimuli of 500 ms length of fixed frequency, varying between conditions: 1 (200 Hz, 124 dB), 2 (400 Hz, 126 dB), 3 (600 Hz, 117 dB), 4 (800 Hz, 111 dB). Stimuli were interleaved by ISIs of a fixed duration of 1 s.

#### Study 2b—Effect of the acoustic power on the ASR habituation

The paradigm consisted of a series of 20 vibratory stimuli of 500 ms length of varying amount of power in the 400 Hz frequency: 1 (100 dB), 2 (108 dB), 3 (118 dB), 4 (126 dB). Stimuli were again interleaved by ISIs of a fixed duration of 1 s.

#### Study 2c—Effect of ISI duration on the ASR habituation

The paradigm consisted of a series of 20 vibratory stimuli of 500 ms length of varying amount of fixed frequency (400 Hz) and power (126 dB), interleaved by ISIs whose duration differed between conditions: 1 (500 ms), 2 (1 s), 3 (3 s), 4 (5 s).

#### Study 3—Effect of light-flash illuminance level on the VSR habituation

The paradigm consisted of a series of 20 white light-flashes of 500 ms length, of fixed illuminance, different in three experimental conditions: 1 (+ 135 lx), 2 (+ 180 lx), 3 (+ 240 lx). Stimuli were interleaved by ISIs of a fixed duration of 1 s, where illuminance decreased to baseline (environmental) levels (~ 330 lx).

### Experimental procedure

Zebrafish larvae of 4–5 dpf were used for all the experiments. Light exposure of the fish in the ZebraBox during all testings was maintained at environmental levels (M = 330 lx) in order to unalter the normal light cycle of the fish and prevent behavioural changes in arousal and motility resulting from sudden changes in illuminance.

#### Study 1—Extinction of ASR habituation memory

Zebrafish larvae (WIK, N = 144) randomly subdivided in six sub-groups of 24-wells plates were tested. All six sub-groups performed the paradigm twice: the first (baseline) and second (re-test). The ‘re-test time’ was manipulated, and two sub-groups received the second test 1 min after the end of the baseline test, other two sub-groups 5 min after and the last two were re-tested 15 min after, for a total of N = 48 in each ‘re-test condition’.

#### Study 2—Effects of vibratory frequency (a), power (b) and ISI duration (c) on the ASR habituation

Zebrafish larvae were randomly distributed in five groups of 24-wells plates (study a: TU, N = 90, 18 wells per plate; studies b and c: WIK, N = 120, 24 wells per plate). Four groups were assigned to one experimental condition, while one performed as control group. The latter was exposed to the same environmental setting as the experimental groups, for the duration of all the experiments, but received no stimulation. Each group commenced the first run of the experiment with one of the four different experimental conditions or control. On each successive run, the group-assignment of a given experimental condition rotated, until all the five groups had performed all the experimental conditions once (total runs = 5). Such counterbalancing the conditions allowed to account for the effect of eventual between-groups differences in age/development and time of testing. For a schematic summary of the experimental design specific of each study. Based on the results of study 1, showing extinction of ASR habituation memory by ¼ h, it was assumed that an inter-run time of ½ h would ensure the full recovery of the fish to baseline-level behaviour. Each run was thus separated by the following run by a pause of 30 min, where the fish would rest in the incubator. Because of the differences in ISIs, the duration of each of the five runs in study 2c differed between conditions, and lasted 50 s, 60 s, 1 min 40 s, 2 min 20 s for the 500 ms, 1 s, 3 s, 5 s ISI duration conditions, respectively.

#### Study 3—Effect of light-flash illuminance level on the VSR habituation

Zebrafish larvae (WIK, N = 96) were randomly distributed in four 24-well plates. The plates/groups were randomly assigned to either an illuminance-condition (three experimental) or to the control condition. Each experimental condition was performed once at each run (tot runs = 4), with the order of start being counterbalanced, in the same way as for study 4.

### Behavioural tracking and quantitative modelling

The fish movements were tracked by mean of the Zebralab software and Zebrabox apparatus, two technologies for behavioural monitoring analysis by Viewpoint Life Sciences (Lyon, France). The Zebrabox speaker, designed and assembled by Viewpoint, was placed at a distance of 11 cm relative to the centre of the well and it was used, together with a commercial amplifier (CS-PA 1 MK, Dynavox), to produce side-wise vibratory stimuli of given power [dB] and frequency [Hz]. The light source in the VSR experiment (study 4) was a rectangular LED, which emitted white whole-field light flashes from beneath the wells.

Startle responses were usually quantified in terms of total distance travelled (continuous measure [mm]) and count of responses > 2 mm (discrete measure), occurring within the duration of each single stimulus (500 ms). This means, the movements occurring during the ISIs were not quantified. The CDT was quantified as the mean total cumulative distance travelled at each stimulus (tot = 20) and the average CDT ranks were statistically assessed for differences between the experimental conditions in all studies. The probability of response, defined as the percentage of total fish that reacted to the first stimulus, was calculated for each experimental condition, in each study. For all the studies, all fish that performed the experiments were included in the analysis (i.e., no exclusion criteria).

**Figure 7:**
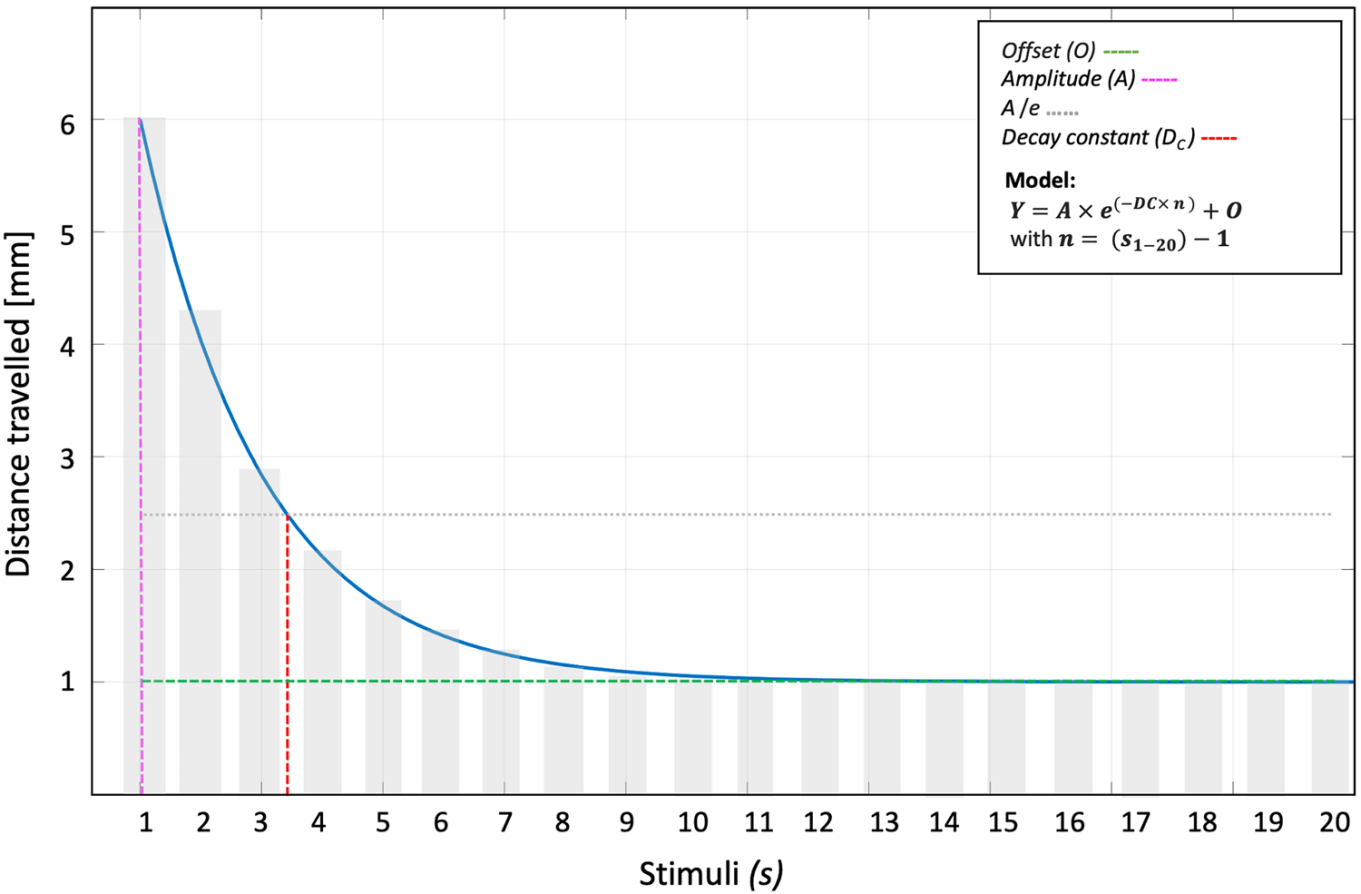
First-order exponential-fitting model used to describe the decay constant of startle habituation.

Data bootstrapping (N = 250) was performed on the distance travelled at each stimulus, by each individual fish. Such bootstrapping analysis, describing the behaviour at population level, was applied for every experimental condition in all three studies. A first-order exponential was fitted into the bootstrapped data to characterise the startle reflex habituation. The model is exposed in Fig. 7. Three different parameters were extracted from the fitted data.

- *Offset (O)* The long-term steady-state response value, namely the responsivity-level of a fish after habituation has occurred. It is obtained as the average distance travelled in response to the last stimuli and corresponds to the steady-state responsivity level.
- *Amplitude (A)* Measure of individual-fish ‘vigorousness’ or magnitude of startle reflex, defined as distance travelled in response to the first stimulus.
- *Decay constant (D*_*C*_*)* Time required for the amplitude to fall to 1/e (~ 36.8%) of its initial value.

### Statistical analyses

Statistical analyses were conducted using MATLAB R2019b (The MathWorks Inc., Natick, Massachusetts, USA) and SPSS Statistics version 25.0 (IBM Corp., Armonk, New York, USA). As the startle responses (mean CDT values) had a non-Gaussian distribution, as verified in previous zebrafish^74^ and human^75^ startle research, non-parametric tests were chosen to assess between-groups differences in average CDT ranks during all 20 stimuli for each study. Wilcoxon signed-rank tests were performed to assess eventual significant differences between the baseline and re-test median CDT ranks for each of the three experimental groups (1, 5, 15 min re-test). Friedman’s tests were run for assessing statistical differences in the mean CDT ranks between the experimental conditions in each study. Finally, Spearman’s rank correlation tests were run to assess the association between two measures: mean count of responses and mean distance travelled, for each frequency condition.

## References

[1] Painter CA, Ceol CJ, Wajapeyee N (2014). Zebrafish as a platform to study tumor progression. Cancer Genomics and Proteomics. Methods in Molecular Biology (Methods and Protocols).

[2] Westerfield M (2007). The Zebrafish Book: A Guide for the Laboratory Use of Zebrafish (*Danio rerio*).

[3] Choo BKM, Shaikh MF, Bozkurt Y (2018). Zebrafish model of cognitive dysfunction. Recent Advances in Zebrafish Researches.

[4] Fleisch VC, Fraser B, Allison WT (2011). Investigating regeneration and functional integration of CNS neurons: lessons from zebrafish genetics and other fish species. Biochimica et Biophysica Acta (BBA) Mol. Basis Disease.

[5] Guo S (2009). Using zebrafish to assess the impact of drugs on neural development and function. Expert Opin. Drug Discov..

[6] Hildebrand DGC (2017). Whole-brain serial-section electron microscopy in larval zebrafish. Nature.

[7] Saleem S, Kannan RR (2018). Zebrafish: an emerging real-time model system to study Alzheimer’s disease and neurospecific drug discovery. Cell Death Discov..

[8] Rihel J, Ghosh M, Hock F (2016). Zebrafish. Drug Discovery and Evaluation: Pharmacological Assays.

[9] Bhandiwad AA, Raible DW, Rubel EW, Sisneros JA (2018). Noise-induced hypersensitization of the acoustic startle response in larval zebrafish. J. Assoc. Res. Otolaryngol..

[10] Nicolson T (1998). Genetic analysis of vertebrate sensory hair cell mechanosensation: The zebrafish circler mutants. Neuron.

[11] Lu Z, Tomchik S (2002). Effects of a red-tide toxin on fish hearing. J. Comp. Physiol. A..

[12] Ladich F, Fay RR (2013). Auditory evoked potential audiometry in fish. Rev. Fish Biol. Fisheries.

[13] Yao Q, DeSmidt AA, Tekin M, Liu X, Lu Z (2016). Hearing assessment in zebrafish during the first week postfertilization. Zebrafish.

[14] Liu X, Lin J, Zhang Y, Guo N, Li Q (2018). Sound shock response in larval zebrafish: A convenient and high-throughput assessment of auditory function. Neurotoxicol. Teratol..

[15] Schlege DK, Neuhauss SC, Gerlai R (2020). The larval visual system and behavioral responses to visual stimuli. Behavioral and Neural Genetics of Zebrafish.

[16] Scheetz SD (2018). An open-source method to analyze optokinetic reflex responses in larval zebrafish. J. Neurosci. Methods.

[17] Colwill RM, Creton R (2011). Imaging escape and avoidance behavior in zebrafish larvae. Rev. Neurosci..

[18] Gibb AC, Swanson BO, Wesp H, Landels C, Liu C (2006). Development of the escape response in teleost fishes: Do ontogenetic changes enable improved performance?. Physiol. Biochem. Zool..

[19] Bang PI, Yelick PC, Malicki JJ, Sewell WF (2002). High-throughput behavioral screening method for detecting auditory response defects in zebrafish. J. Neurosci. Methods.

[20] Kimmel CB, Patterson J, Kimmel RO (1974). The development and behavioral characteristics of the startle response in the zebrafish. Dev. Psychobiol..

[21] Burgess HA, Granato M (2007). Modulation of locomotor activity in larval zebrafish during light adaptation. J. Exp. Biol..

[22] Issa FA (2011). Neural circuit activity in freely behaving zebrafish (*Danio rerio*). J. Exp. Biol..

[23] Baillargeon A, Lassonde M, Leclerc S, Ellemberg D (2012). Neuropsychological and neurophysiological assessment of sport concussion in children, adolescents and adults. Brain Inj..

[24] Eaton RC, Bombardieri RA, Meyer DL (1977). The Mauthner-initiated startle response in teleost fish. J. Exp. Biol..

[25] Medan V, Preuss T (2014). The Mauthner-cell circuit of fish as a model system for startle plasticity. J. Physiol.-Paris.

[26] Korn H, Faber DS (2005). The Mauthner cell half a century later: A neurobiological model for decision-making?. Neuron.

[27] Muto A (2017). Activation of the hypothalamic feeding centre upon visual prey detection. Nat. Commun..

[28] Roberts AC (2011). Habituation of the C-start response in larval zebrafish exhibits several distinct phases and sensitivity to NMDA receptor blockade. PLoS ONE.

[29] López-Schier H (2019). Neuroplasticity in the acoustic startle reflex in larval zebrafish. Curr. Opin. Neurobiol..

[30] Pantoja C (2016). Neuromodulatory regulation of behavioral individuality in zebrafish. Neuron.

[31] Park C, Clements KN, Issa FA, Ahn S (2018). Effects of social experience on the habituation rate of zebrafish startle escape response: Empirical and computational analyses. Front. Neural Circ..

[32] Koch M (1999). The neurobiology of startle. Prog. Neurobiol..

[33] Kehne JH, Boulis NM, Davis M (1991). Effects of the phosphodiesterase inhibitor rolipram on the acoustic startle response in rats. Psychopharmacology.

[34] Klamer D, Pålsson E, Revesz A, Engel JA, Svensson L (2004). Habituation of acoustic startle is disrupted by psychotomimetic drugs: Differential dependence on dopaminergic and nitric oxide modulatory mechanisms. Psychopharmacology.

[35] Bhandiwad AA, Zeddies DG, Raible DW, Rubel EW, Sisneros JA (2013). Auditory sensitivity of larval zebrafish (*Danio rerio*) measured using a behavioral prepulse inhibition assay. J. Exp. Biol..

[36] Takahashi H (2011). Prepulse inhibition of startle response: Recent advances in human studies of psychiatric disease. Clin. Psychopharmacol. Neurosci..

[37] Rankin CH (2009). Habituation revisited: An updated and revised description of the behavioral characteristics of habituation. Neurobiol. Learn. Mem..

[38] Roberts AC (2016). Long-term habituation of the C-start escape response in zebrafish larvae. Neurobiol. Learn. Mem..

[39] Cho W, Heberlein U, Wolf FW (2004). Habituation of an odorant-induced startle response in Drosophila. Genes Brain Behav..

[40] Marcus EA, Nolen TG, Rankin CH, Carew TJ (1988). Behavioral dissociation of dishabituation, sensitization, and inhibition in Aplysia. Science.

[41] Pletnicov MV, Storozheva ZI, Sherstnev VV (1995). Developmental analysis of habituation of the acoustic startle response in the preweanling and adult rats. Behav. Proc..

[42] Brown GD (1998). Nonassociative learning processes affecting swimming probability in the seaslug Tritonia diomedea: Habituation, sensitization and inhibition. Behav. Brain Res..

[43] Eaton RC, Farley RD, Kimmel CB, Schabtach E (1977). Functional development in the Mauthner cell system of embryos and larvae of the zebra fish. J. Neurobiol..

[44] Takahashi H, Kamio Y (2018). Acoustic startle response and its modulation in schizophrenia and autism spectrum disorder in Asian subjects. Schizophr. Res..

[45] Liska GM, Lee JY, Xu K, Sanberg PR, Borlongan CV (2018). Suppressed acoustic startle response in traumatic brain injury masks post-traumatic stress disorder hyper-responsivity. NeuroReport.

[46] Pang KC (2015). Long-lasting suppression of acoustic startle response after mild traumatic brain injury. J. Neurotrauma.

[47] Takahashi M, Inoue M, Tanimoto M, Kohashi T, Oda Y (2017). Short-term desensitization of fast escape behavior associated with suppression of Mauthner cell activity in larval zebrafish. Neurosci. Res..

[48] Best JD (2008). Non-associative learning in larval zebrafish. Neuropsychopharmacology.

[49] Higgs DM, Souza MJ, Wilkins HR, Presson JC, Popper AN (2002). Age-and size-related changes in the inner ear and hearing ability of the adult zebrafish (*Danio rerio*). JARO J. Assoc. Res. Otolaryngol..

[50] Gerlai R (2019). Reproducibility and replicability in zebrafish behavioral neuroscience research. Pharmacol. Biochem. Behav..

[51] Ulrich F, Grove C, Torres-Vázquez J, Baker R (2016). Development of functional hindbrain oculomotor circuitry independent of both vascularization and neuronal activity in larval zebrafish. Curr. Neurobiol..

[52] Portugues R, Engert F (2011). Adaptive locomotor behavior in larval zebrafish. Front. Syst. Neurosci..

[53] Quon EF, Wotton CA, Bekar LK (2018). Evidence for astrocyte purinergic signaling in cortical sensory adaptation and serotonin-mediated neuromodulation. Mol. Cell. Neurosci..

[54] Wong WM (2018). Sensory adaptation to chemical cues by vomeronasal sensory neurons. eNeuro.

[55] Graczyk EL, Delhaye BP, Schiefer MA, Bensmaia SJ, Tyler DJ (2018). Sensory adaptation to electrical stimulation of the somatosensory nerves. J. Neural Eng..

[56] Hewson L, Tarrega A, Hort J, Kemp S, Hollowood T (2017). Sensory adaptation. Time-Dependent Measures of Perception in Sensory Evaluation.

[57] Wark B, Lundstrom BN, Fairhall A (2007). Sensory adaptation. Curr. Opin. Neurobiol..

[58] Stocker AA, Simoncelli EP, Weiss Y, Scholkopf B, Platt J (2005). Sensory adaptation within a Bayesian framework for perception. Advances in Neural Information Processing Systems: Proceedings of the 18th International Conference on Neural Information Processing Systems.

[59] Castellucci V, Pinsker H, Kupfermann I, Kandel ER (1970). Neuronal mechanisms of habituation and dishabituation of the gill-withdrawal reflex in Aplysia. Science.

[60] Groves PM, Thompson RF (1970). Habituation: A dual-process theory. Psychol. Rev..

[61] Levy RM (2019). Therapy habituation at 12 months: Spinal cord stimulation versus dorsal root ganglion stimulation for complex regional pain syndrome type I and II. J. Pain..

[62] Davis M, Parisi T, Gendelman DS, Tischler M, Kehne JH (1982). Habituation and sensitization of startle reflexes elicited electrically from the brainstem. Science.

[63] Schmitz V, Renou M, Roehrich R, Stockel J, Lecharpentier P (1997). Disruption mechanisms of pheromone communication in the European grape moth Lobesia botrana Den & Schiff. III. Sensory adaptation and habituation. J. Chem. Ecol..

[64] Kuenen LPS, Baker TC (1981). Habituation versus sensory adaptation as the cause of reduced attraction following pulsed and constant sex pheromone pre-exposure in Trichoplusia ni. J. Insect Physiol..

[65] Brunner D, Maldonado H (1988). Habituation in the crab *Chasmagnathus granulatus*: Effect of morphine and naloxone. J. Comp. Physiol. A..

[66] Baglan H, Lazzari C, Guerrieri F (2017). Learning in mosquito larvae (*Aedes aegypti*): Habituation to a visual danger signal. J. Insect Physiol..

[67] Larkin A (2010). Central synaptic mechanisms underlie short-term olfactory habituation in *Drosophila larvae*. Learn. Memory.

[68] Pellegrino R, Sinding C, De Wijk RA, Hummel T (2017). Habituation and adaptation to odors in humans. Physiol. Behav..

[69] Girdhar K, Gruebele M, Chemla YR (2015). The behavioral space of zebrafish locomotion and its neural network analog. PLoS ONE.

[70] Budick SA, O'Malley DM (2000). Locomotor repertoire of the larval zebrafish: Swimming, turning and prey capture. J. Exp. Biol..

[71] Bollmann JH (2019). The zebrafish visual system: From circuits to behavior. Annu. Rev. Vis. Sci..

[72] Haug MF, Biehlmaier O, Mueller KP, Neuhauss SC (2010). Visual acuity in larval zebrafish: Behavior and histology. Front. Zool..

[73] Kalueff AV (2013). Towards a comprehensive catalog of zebrafish behavior 1.0 and beyond. Zebrafish.

[74] Miller, E. A., Kastner, D. B., Grzybowski, M. N., Dwinell, M. R., Geurts, A. M. & Frank, L. M. Robust and replicable measurement for prepulse inhibition of the acoustic startle response. *bioRxiv*10.1101/601500 (2020). 10.1038/s41380-020-0703-y PMC7483293 32144356

[75] Bakker MJ, Tijssen MA, van der Meer JN, Koelman JH, Boer F (2009). Increased whole-body auditory startle reflex and autonomic reactivity in children with anxiety disorders. J. Psychiatry Neurosci..

