# Author Correction: A model-based quantification of startle reflex habituation in larval zebrafish

**DOI:** 10.1038/s41598-021-87654-5

**Published:** 2021-04-08

**Authors:** Carolina Beppi, Dominik Straumann, Stefan Yu Bögli

**Affiliations:** 1 Neuroscience Center Zurich, University of Zurich and ETH Zurich, CH-8091 Zurich, Switzerland; 2 Department of Neurology, University Hospital Zurich and University of Zurich, CH-8091 Zurich, Switzerland; 3 Clinical Neuroscience Center, University Hospital Zurich and University of Zurich, CH-8091 Zurich, Switzerland; 4 Swiss Concussion Center, Schulthess Clinic, CH-8008 Zurich, Switzerland

Correction to: *Scientific Reports* https://doi.org/10.1038/s41598-020-79923-6, published online 12 January 2021

This Article contains errors in Figure 3, where the figure panels A–E present incorrect x-axis labels.

The correct version of Figure 3 appears below as Figure 1.

**Figure 1:**
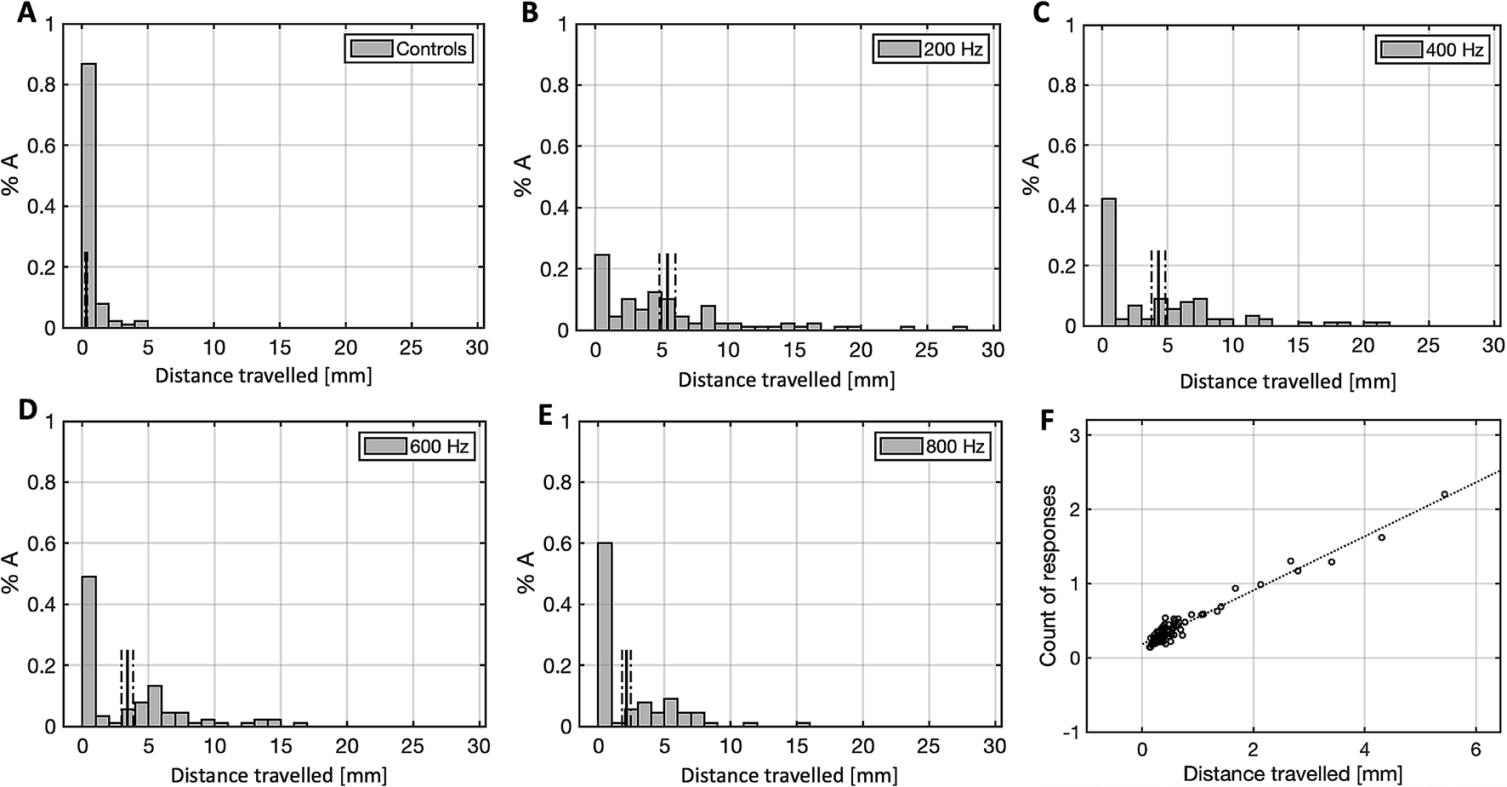
A correct version of the original Figure 3.

